# Implications of depressive mood in OSAHS patients: insights from event-related potential

**DOI:** 10.1186/s12888-024-05772-6

**Published:** 2024-04-23

**Authors:** Zhiqiang Li, Sijie Cai, Jiamin Qiao, Yezhou Li, Qiaojun wang, Rui Chen

**Affiliations:** 1 Department of Respiratory and Critical Care Medicine, Sleep Center, The Second Affiliated Hospital of Soochow University, Suzhou, China; 2 Department of Pulmonary and Critical Care Medicine, Affiliated Kunshan Hospital of Jiangsu University, Suzhou, China; 3 Oxford University Clinical Academic Graduate School and Buckinghamshire Healthcare NHS Trust, Oxford, UK

**Keywords:** Obstructive sleep apnea-hypopnea syndrome, Depressive mood, Facial emotion recognition, Event-related potential

## Abstract

**Background:**

Obstructive sleep apnea-hypopnea syndrome (OSAHS) is a chronic breathing disorder characterized by recurrent upper airway obstruction during sleep. Although previous studies have shown a link between OSAHS and depressive mood, the neurobiological mechanisms underlying mood disorders in OSAHS patients remain poorly understood. This study aims to investigate the emotion processing mechanism in OSAHS patients with depressive mood using event-related potentials (ERPs).

**Methods:**

Seventy-four OSAHS patients were divided into the depressive mood and non-depressive mood groups according to their Self-rating Depression Scale (SDS) scores. Patients underwent overnight polysomnography and completed various cognitive and emotional questionnaires. The patients were shown facial images displaying positive, neutral, and negative emotions and tasked to identify the emotion category, while their visual evoked potential was simultaneously recorded.

**Results:**

The two groups did not differ significantly in age, BMI, and years of education, but showed significant differences in their slow wave sleep ratio (*P* = 0.039), ESS (*P* = 0.006), MMSE (*P* < 0.001), and MOCA scores (*P* = 0.043). No significant difference was found in accuracy and response time on emotional face recognition between the two groups. N170 latency in the depressive group was significantly longer than the non-depressive group (*P* = 0.014 and 0.007) at the bilateral parieto-occipital lobe, while no significant difference in N170 amplitude was found. No significant difference in P300 amplitude or latency between the two groups. Furthermore, N170 amplitude at PO7 was positively correlated with the arousal index and negatively with MOCA scores (both *P* < 0.01).

**Conclusion:**

OSAHS patients with depressive mood exhibit increased N170 latency and impaired facial emotion recognition ability. Special attention towards the depressive mood among OSAHS patients is warranted for its implications for patient care.

**Supplementary Information:**

The online version contains supplementary material available at 10.1186/s12888-024-05772-6.

## Introduction

Obstructive sleep apnea-hypopnea syndrome (OSAHS) is a chronic breathing disorder, characterized by recurrent episodes of partial or complete obstruction of the upper respiratory airways during sleep, leading to lack of nocturnal oxygen saturation and micro-awakenings [1]. Patients with OSAHS experience hypopnea and apnea repeatedly during the night resulting in systemic damage. In some cases, OSAHS can progress towards anxiety, depression, and Alzheimer’s disease, mediated by OSA-induced structural and functional abnormalities of the brain [2, 3].

Epidemiological studies had consistently shown a link between OSAHS and depression [4–7]. A meta-analysis including 73 studies has reported that the prevalence of depressive symptoms in patients with OSAHS was up to 35% [8]. In a cross-sectional study including 18,980 subjects, patients with major depressive disorder were five times more likely to have OSAHS than healthy individuals. A study from 1998 to 2001 in the Veterans Health Administration database showed that the prevalence of mood disorders in OSAHS patients was 21.75%, significantly higher than in non-OSAHS patients, at 9.43% [9]. Furthermore, two longitudinal studies demonstrated that OSAHS is an independent risk factor for depression, with a 2 to 2.6-fold increase in the odds of developing depression in patients with moderate to severe OSAHS [6, 7].

Patients with OSAHS frequently experience mood disorders and cognitive impairments. Several potential mechanisms contribute to these conditions, such as intermittent hypoxia, hypercapnia, sleep fragmentation, and reduced cerebral blood flow. These mechanisms can activate the infiltration of oxidative stress products and inflammatory cells into the central nervous system, consequentially leading to functional disruptions, pathological changes, and even structural abnormalities. Ultimately, these factors contribute to mood disorders and neurocognitive dysfunction in individuals with OSAHS [10–13]. However, the underlying neuro-electrophysiological mechanisms remain to be fully understood to design effective interventions.

Event-related potentials (ERP) is a task-specific brain-evoked potential, whose amplitude and latency can reflect the neuro-electrophysiological changes of emotional and cognitive processing at the neuronal level [14]. ERPs with a high temporal resolution can reveal the temporal dynamics of disrupted information processing more objectively than traditional subjective scale tests [15]. Patients with OSAHS could show impairments in several cognitive domains including attention and memory as well as mood disorders including depression and anxiety [16]. A recent study had shown that patients with OSAHS had prolonged latency of the P300 ERP component, potentially suggesting dysfunctional information processing and attention [17]. Another ERP study by Wen et al. (*N* = 97) found a significant correlation between the prolonged latency of the mismatch negativity (MMN) in a classical oddball paradigm among OSAHS patients compared to controls, and the decline in Montreal Cognitive Assessment (MOCA) scores [18]. As such, ERP analysis has proven to be an effective tool in the assessment of cognitive abilities in OSAHS patients, providing unique insights into the potential mechanism of cognitive processes beyond that of behavioral measures. However, Previous ERP studies on OSAHS have predominantly focused on attention and memory function [17, 18], while the neurobiological mechanisms underlying OSAHS-related mood disorders remain scarcely researched. In this study, we used the emotional face ERP paradigm to examine the emotion processing mechanism in OSAHS patients combined with depressive mood to potentially provide new insights to improve the emotional well-being of patients.

## Materials and methods

### Subjects

**Fig. 1:**
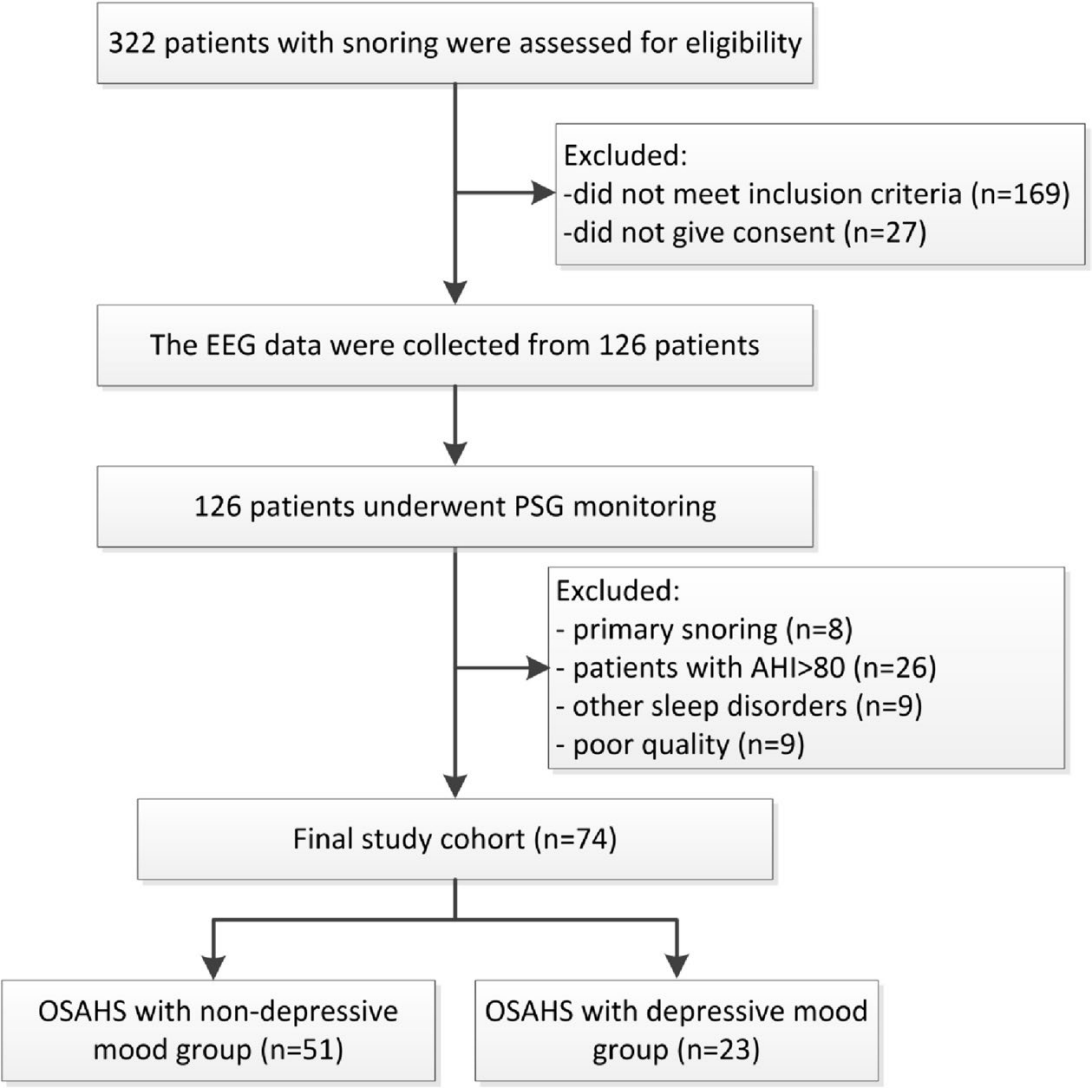
Screening process of patient selection and inclusion

This prospective study was conducted at the Sleep Center of the Second Affiliated Hospital of Soochow University. From August 2021 to August 2022, male patients received at the sleep center with a complaint of snoring and underwent overnight polysomnography (PSG) monitoring to be diagnosed with OSAHS were screened for their eligibility for the study. All patients were aged 18–60 years, with secondary education or above. Patients with extremely severe OSAHS with the apnea-hypopnea index (AHI > 80) events/h and severe obesity with BMI > 35 kg/m² were excluded. Patients have not undergone CPAP or other interventions for OSAHS. Patients with psychiatric, neurological disorders (e.g., insomnia, Alzheimer’s disease), sleep disorders (e.g., central sleep apnea, sleep movement disorders), or other serious diseases (e.g., heart failure, stroke history) were excluded. 74 patients were included in the final study cohort (Fig. 1). Notably, only male patients were included in the study, as previous large-sample studies found that the clinical phenotype of female patients with OSAHS differs from that of male patients [19, 20].

### Clinical assessment

The patients were assessed in terms of daytime sleepiness, sleep quality, emotional states of depression and anxiety, and cognitive function using the Epworth Sleep Scale (ESS) [21], the Pittsburgh Sleep Quality Index Scale (PSQI) [22], the Mini-Mental Status Examination (MMSE) [23], Montreal Cognitive Assessment (MOCA) [24], Depression Self-assessment Scale (SDS) [25], and Anxiety Self-assessment Scale (SAS) [26]. These assessments were conducted in the morning on the day following the conclusion of polysomnography (PSG) testing, with the goal of minimizing potential fatigue-related influences on the results. The SDS questionnaire contains 20 items assessing self-reported frequency of depressive symptoms in the past week, on a 4-point scale, totaling 80 points. The raw score, multiplied by 1.25, was transformed into a standard score ranging from 25 to 100 points. Despite recent research [27] suggesting that a raw value of 50 is more effective for diagnosing depression, our utilization of the SDS scale is specifically geared toward assessing depressive mood in OSAHS patients, rather than clinical depression. Therefore, we have chosen to use to raw value of 40 as the cutoff score. By applying an SDS standard score cutoff of 50 points, patients were categorized into either the depressive mood group or the non-depressive mood group [25]. The study was approved by the Soochow University ethics committee, and all patients gave informed consent.

### Polysomnography monitoring

All participants underwent PSG monitoring. Patients were advised to avoid consuming tea, caffeine, alcohol, sedatives, and hypnotics on the day. Effective PSG recording time was greater than seven hours for all patients. PSG recordings included eight EEG channels: frontal (F3 and F4), central (C3 and C4), occipital (O1 and O2), and reference electrode (M1 and M2) based on the standard 10–20 system; electrooculography, maxillofacial electromyography, electrocardiography, temperature-sensitive, oral and nasal respiratory airflow, snoring, chest and abdomen breathing movement, and body position, blood oxygen saturation, and leg movement. The signal acquisition for all participants was carried out using the Compumedics Grael multifunctional PSG monitoring system from Compumedics in Australia. Subsequent to the data acquisition, sleep staging and sleep-related respiratory analyses were meticulously conducted manually by a registered technician. These analyses adhered to the established criteria outlined by the American Academy of Sleep Medicine (AASM) [28]. The apnea-hypopnea index (AHI) was computed as the average number of apneas and hypopneas per hour of sleep, considering instances with a desaturation of ≥ 3% or an arousal. Concurrently, the oxygen desaturation index was defined as the mean number of events with a ≥ 3% desaturation per hour of sleep.

### Task-state EEG data acquisition

**Fig. 2:**
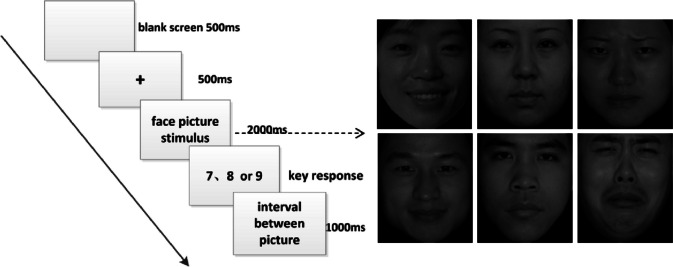
The experimental design schematic of the emotional face ERP paradigm

Patients participated in an EEG-recorded facial emotion recognition task the morning before their polysomnography. 180 facial images displaying one of positive, negative, and neutral emotions (60 each) were selected from the Chinese Affective Picture System (CAPS) [29]. The experiment was programmed with the E-Prime 2.0 software. The experiment included two stages, a practice stage, and an experimental stage. In the practice stage, participants were given six example trials with two trials in each condition. To prevent the practice effect from causing memory biases during the formal experiment, the images used in the practice program was not be included in the formal experiment. The formal experiment was as follows: Instruction - Blank screen 500ms - fixation point (+) 500ms - face picture stimulus 2000ms - key response (number keys “7”, “8” and “9” on the computer keyboard, each corresponding to positive, neutral, negative emotions, respectively). The interval between each picture is 1000ms. A schematic of the stimulus design is shown in Fig. 2.

### EEG data analysis and statistical methods

 Preprocessing of EEG data was performed by the EEGLAB toolbox with MATLAB 2013b. (1) the recording was down-sampled to 250 Hz; (2) band-pass filtering was set to 0.5–30 Hz; (3) the whole brain average value was used for re-referencing; (4) independent component analysis (ICA) was performed to remove non-brain-derived signal; (5) we selected the PO7 (left parieto-occipital lobe) and PO8 electrodes (right parieto-occipital lobe) to analyze the ERP amplitude and latency. SPSS 22.0 was used for statistical analysis. Normally distributed variables were presented using the mean and standard deviation, whereas non-normally distributed variables were presented as median with interquartile range (IQR). Two-sample independent t-tests were used for comparison between the two groups, and correlation analysis was performed using Pearson’s correlation test. Correlation plots and box plots were produced using GraphPad 9.0.

## Results

### Baseline demographic and clinical characteristics

The demographic and clinical characteristics are summarized in Table 1. The OSAHS patients were divided into two groups according to their SDS scores: The non-depressive mood group (SDS score < 50) and the depressive mood group (SDS score > = 50). No significant differences were observed in age, BMI, and level of education between the two groups, but significant differences were shown in SWS% (11.4(4.9–18.2) vs. 6.3(2.3–11.6), *P* = 0.039), ESS (6.0(4.0–11.0) vs. 12.0(6.0–16.0), *P* = 0.006), MMSE (10.95 ± 6.07 vs. 28.23 ± 1.69, *P* < 0.001), MOCA (26.87 ± 2.27 vs. 25.52 ± 3.07, *P* = 0.043) and SAS (38.08 ± 7.36 vs. 43.63 ± 7.39, *P* = 0.004) between the two groups.

**Table 1:** Demographic and Clinical Characteristics of the OSAHS patients with depressive mood and non-depressive mood

SDS categorical	< 50 (N = 51)	>=50 (N = 23)	Standardize diff.	P-value
Baseline demographic Characteristics				
AGE (years)	39.5 ± 6.8	43.2 ± 10.6	0.4 (-0.1, 0.9)	0.125
BMI (kg/m^2^)	26.7 ± 3.2	27.8 ± 3.6	0.3 (-0.2, 0.9)	0.211
EDUCATION (years)	14.9 ± 3.5	13.1 ± 3.4	0.5 (-0.1, 1.1)	0.088
PSG parameters				
TST (min)	423.2 ± 92.4	438.5 ± 63.0	0.2 (-0.3, 0.7)	0.485
Sleep Efficiency (%)	86.3 ± 12.0	85.8 ± 11.5	0.0 (-0.5, 0.5)	0.866
Sleep Latency (min)	3.2(1.5-6.0)	4.8(2.0-5.9)	0.2 (-0.3, 0.7)	0.564
AROUSE (N/hr)	23.0(17.8–36.5)	35.5(18.8–43.0)	0.3 (-0.2, 0.9)	0.148
N1 (%)	14.0(7.5–21.8)	13.0(9.9–23.0)	0.2 (-0.3, 0.7)	0.407
N2 (%)	53.0 ± 11.2	56.4 ± 11.3	0.3 (-0.2, 0.8)	0.238
REM (%)	19.1 ± 5.4	18.2 ± 4.7	0.2 (-0.3, 0.7)	0.510
SWS (%)	11.4(4.9–18.2)	6.3(2.3–11.6)	0.6 (0.1, 1.1)	0.039*
ODI (N/hr)	30.0(9.7–54.0)	27.8(10.3–66.1)	0.3 (-0.2, 0.8)	0.262
TS90 (%)	7.1(0.9–19.6)	13.5(2.5–33.4)	0.3 (-0.2, 0.8)	0.198
minSaO2 (%)	73.3 ± 13.5	69.9 ± 15.8	0.2 (-0.3, 0.7)	0.348
AHI (N/hr)	39.0(21.6–57.5)	40.0(15.3–71.0)	0.2 (-0.3, 0.7)	0.444
Sleep and cognitive scale				
PSQ	5.3 ± 2.8	5.9 ± 3.0	0.18 (-0.4, 0.7)	0.499
ESS	6.0(4.0–11.0)	12.0(6.0–16.0)	0.70 (0.2, 1.2)	0.006**
MMSE	29.4 ± 1.0	28.2 ± 1.7	0.83 (0.3, 1.4)	< 0.001**
MOCA	26.9 ± 2.3	25.5 ± 3.1	0.5 (-0.0, 1.0)	0.043*
SAS	38.1 ± 7.4	43.6 ± 7.4	0.8 (0.3, 1.3)	0.004**
SDS	40.5 ± 6.2	56.3 ± 5.3	NA	NA
HYPERTENSION	24 (47.1%)	15 (65.2%)	0.4 (-0.1, 0.9)	0.148
Bedtime SBP (mmHg)	131.0 ± 16.3	135.1 ± 18.5	0.24 (-0.3, 0.8)	0.349
Morning SBP (mmHg)	131.1 ± 18.9	137.3 ± 12.4	0.4 (-0.2, 1.0)	0.224
Bedtime DBP (mmHg)	91.0 ± 14.9	90.6 ± 11.6	0.0 (-0.5, 0.5)	0.900
Morning DBP (mmHg)	92.9 ± 13.3	96.4 ± 14.9	0.3 (-0.3, 0.8)	

*TST *Total sleep time, *REM *Rapid eye movement, *SWS *Slow wave sleep, *ODI *Oxygen desaturation index, *TS90 *percentage of total sleep time at oxygen saturation level < 90%, *minSaO2 *lowest arterial oxygen saturation, *AROUSE *Arousal index, *SBP *Systolic blood pressure, *DBP *Diastolic blood Pressure

* *p* < 0.05

** *p* < 0.01

### Behavioral parameters

There was no significant statistical difference in the reaction time and accuracy between OSAHS patients with and without depressive mood. Notably, the comparison of reaction times in recognizing positive emotions between the two groups showed a tendency towards a significant statistical difference (929.1 ± 141.3ms vs. 881.4 ± 114.8ms, *P* = 0.131), potentially indicating a subtle decrease in the inclination towards positive emotions in the group with depressive mood (Table 2).

**Table 2:** Accuracy and reaction time for emotional faces in OSAHS patients with depressive mood and non-depressive mood

SDS categorical	< 50 (*N* = 51)	>=50 (*N* = 23)	Standardize diff.	*P*-value
RT (ms)	897.2 ± 114.4	927.5 ± 128.0	0.2 (-0.2, 0.7)	0.316
RTPOSITIVE (ms)	881.4 ± 114.8	929.1 ± 141.3	0.4 (-0.1, 0.9)	0.131
RTNEUTRAL (ms)	883.6 ± 137.7	904.3 ± 134.9	0.2 (-0.3, 0.6)	0.551
RTNEGATIVE (ms)	924.5 ± 124.7	931.6 ± 104.6	0.1 (-0.4, 0.6)	0.813
ACC (%)	92.3 ± 4.6	90.6 ± 5.1	0.4 (-0.2, 0.9)	0.168
ACCPOSITIVE (%)	91.9 ± 6.9	89.1 ± 8.0	0.4 (-0.1, 0.9)	0.136
ACCNEUTRAL (%)	96.0 ± 4.3	94.3 ± 6.1	0.3 (-0.2, 0.8)	0.160
ACCNEGATIVE (%)	88.9 ± 9.5	89.0 ± 7.8	0.0 (-0.5, 0.5)	0.949

*N *Numbers, *RT *Reaction time, *ACC *Accuracy

### ERP latency and amplitude

**Fig. 3:**
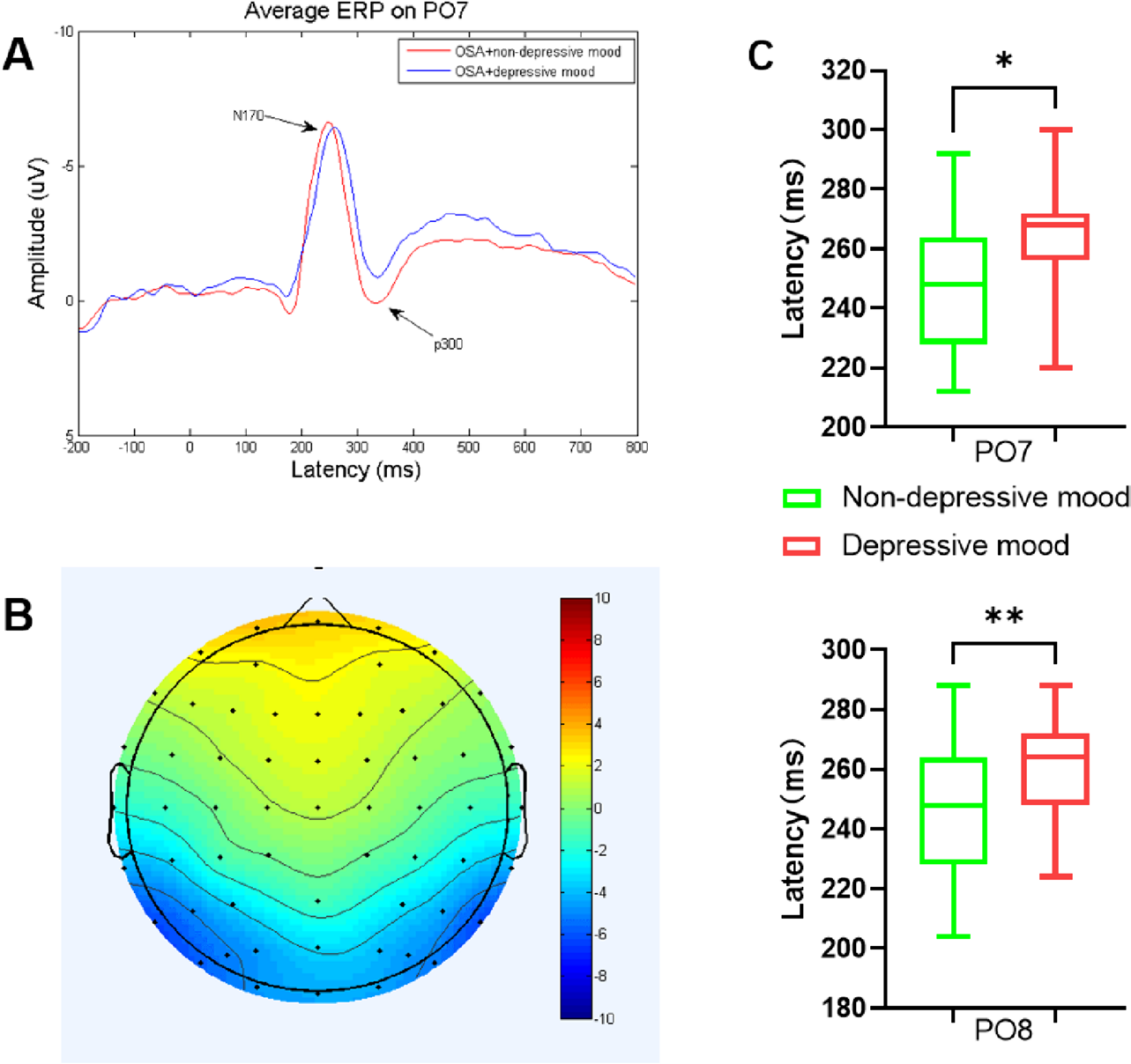
**A **The averaged ERP in patients in OSAHS patients with depressive mood and non-depressive mood at PO7 electrode; (**B**) Topography of the N170; (**C**) Box plot of N170 latency at PO7 and PO8 between two groups; * *p* < 0.05, ** *p* < 0.01

The two representative electrodes of PO7 and PO8 were chosen for the analysis, as these were found to show the most prominent N170 deflections in previous studies [30]. As shown in Fig. 3A and B, the activation of N170 was most prominent in the bilateral parieto-occipital lobe. Visual evoked potential (VEP) exhibited no significant cross-group differences in the amplitude of N170 at PO7 and PO8 (*P* > 0.05), but the latency was significantly prolonged (*P* = 0.017 and 0.006) in patients with depressive mood. There were no significant differences in the amplitude and latency of face-induced P300 at PO7 and PO8 (*P* > 0.05) (Table 3; Fig. 3C).

**Table 3:** ERP amplitude and latency in OSAHS patients with depressive mood and non-depressive mood

SDS categorical	< 50 (*N* = 51)	>=50 (N = 23)	Standardize diff.	*P*-value
PO7.N170L (ms)	248.71 ± 22.41	262.43 ± 22.39	0.61 (0.11, 1.11)	0.014*
PO8.N170L (ms)	246.04 ± 22.98	261.74 ± 20.39	0.72 (0.22, 1.23)	0.007**
PO7.N170A (μV)	-10.05 ± 5.28	-9.16 ± 6.11	0.15 (-0.34, 0.65)	0.329
PO8.N170A (μV)	-9.88 ± 5.51	-7.75 ± 7.26	0.33 (-0.16, 0.83)	0.062
PO7.P300L (ms)	334.75 ± 31.51	338.61 ± 26.90	0.13 (-0.36, 0.63)	0.474
PO8.P300L (ms)	330.12 ± 29.88	343.48 ± 29.05	0.45 (-0.04, 0.95)	0.054
PO7.P300A (μV)	1.70 ± 3.53	0.19 ± 3.45	0.43 (-0.07, 0.93)	0.122
PO8.P300A (μV)	1.68 ± 3.58	1.48 ± 5.87	0.04(-0.45, 0.53)	0.517

*N *Numbers, *L *Latency, *A *Amplitude

* p < 0.05

** p < 0.01

### Relationships between ERP variables and clinical parameters

**Fig. 4:**
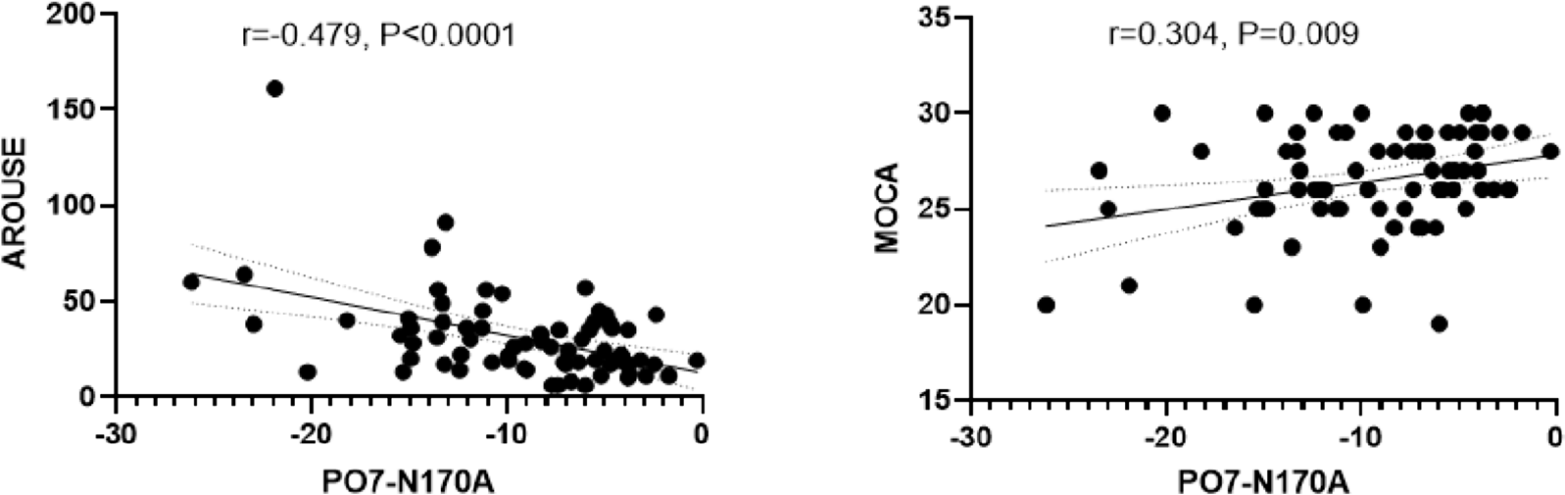
Correlation analysis between N170 amplitude and clinical characteristics in OSAHS patients. RTPositive, Reaction time of positive emotion; N170L, N170 latency; N170A, N170 amplitude

We analyzed the correlations between the N170 parameters (amplitude and latency) and PSG parameters and questionnaire scores (Significant results are shown in Fig. 4). In Fig. 4, the correlation analysis revealed moderate positive associations between the N170 amplitude at PO7 and the arousal index (*r* = 0.479, *P* < 0.0001). Additionally, a weak negative correlation was observed between the N170 amplitude at PO7 and the MOCA scores (*r* = 0.304, *P* = 0.0009).

## Discussion

In this study, we examined the N170 of ERP component in response to emotional faces in OSAHS patients with depressive mood and non-depressive mood and the main findings are as follows: firstly, patients with depressive mood group had lower SWS%, MMSE, and MOCA scores, and higher ESS scores, SAS scores than non-depressive mood group. Secondly, patients with depressive mood exhibited lower difference between negative and positive emotional reaction time, and longer N170 latency than non-depressive mood group. Finally, N170 amplitude at the left occipital lobe showed a negative association with arousal index and a positive association with MOCA score.

The prevalence of depressive mood was reported to be approximately 35% in patients with OSAHS [8]. In our study, despite being of similar age, BMI, level of education, and OSA severity measured by AHI, the patients in the depressive mood group spent less time in slow-wave sleep, reported a higher degree of daytime sleepiness, scored lower on cognitive tests, and complained more symptoms of depression and anxiety. In addition, previous studies have reported that the combination of OSAHS and depression carries heightened risks for metabolic syndrome and cardiovascular diseases [31, 32]. Moreover, depression has been associated with reduced treatment efficacy for OSAHS, further worsening the quality of life [33]. Thus, the OSAHS phenotype overlapping with depressive mood requires increased attention due to its significant implications for patient care, particularly concerning the risks of suicidal ideation associated with depression.

Our results suggested that OSAHS patients with depressive mood had less slow-wave sleep and higher subjective sleepiness. Lee et al. demonstrated that depressive symptoms were associated with sleep fragmentation, not AHI or hypoxia parameters [34] in patients with OSAHS, which is consistent with our study. However, the mechanism for the occurrence of depressive mood is unclear in OSAHS patients. In our study, the depressive mood group had lower cognitive scores than the non-depressive mood group. Similar to our study, Alomri et al. showed that depressive symptoms in OSAHS were independently related to impairment in cognitive domains such as sustained attention, reaction time, visuospatial abilities, and semantic and episodic memory [35]. Furthermore, another study found that a lifetime history of depression was closely linked to increases in the changes of AD-related neuropathological markers within the hippocampus [36]. Therefore, in the group of OSAHS patients already susceptible to cognitive impairment, early identification of depressive symptoms presents opportunities for prompt interventions to delay the progression of cognitive impairment.

To closely examine this depressive phenotype of OSAHS patients, we adapted the emotional face ERP paradigm using images from the Chinese Affective Picture System (CAPS). N170 is regarded as a classic face-specific ERP component that reflects the early perception of facial structures, and is most prominent at the posterior occipitotemporal cortex [30]. Several previous ERP studies have shown that N170 tends to be abnormal in patients with autism, social anxiety disorder, and depression, indicating an impaired ability of emotional regulation [37–39]. Specifically, a systematic review including 67 studies found that, compared to healthy controls, reported changes in N170 were almost always prolonged latency in 13 neurological/psychiatric conditions including major depression [40], in alignment with our findings in the OSAHS patients with depressive mood. Face-processing and recognition-related N170 arises from the parahippocampal cortex in the medial temporal lobe, and the N170 latency effect reflects the degree of theta rhythm activation in the parahippocampal cortex [41, 42]. Notably, studies have revealed a strong correlation between the N170 latency effect and a decline in spatial navigation [43], which itself is a predictor for the development of future dementia in patients with mild cognitive impairment (MCI). As such, increased N170 latency could be a potential biomarker signaling facial recognition defects as well as other cognitive impairments in patients [44].

Interestingly, the behavioral data revealed that comparable reaction times in identifying negative emotions among OSAHS patients with and without depressive mood. However, when comparing reaction times in the recognition of positive emotions, a trend towards statistical difference was observed, with the group experiencing depressive mood tending to exhibit longer reaction times. Although this trend failed to achieve statistical significance, it is noteworthy that a larger sample size might have yielded more conclusive findings. Given the inherent limitations of our study, especially the small sample size, this interpretation must be approached with caution, and we intend to expand the sample size in future studies for further verification. Current psychological research has suggested a connection between depression and the processing of social stimuli. Individuals with depression may struggle to fully experience pleasure derived from others’ praise, acceptance, and goodwill, leading to relatively longer reaction times to positive stimuli. Specifically, abnormalities in emotion recognition, especially recognition defects for positive emotions [45] and recognition preferences for negative emotions [46], may be important causes of negative emotional states. Furthermore, a study by Gobin et al. showed that individuals with poor sleep quality exhibited a similar preference towards negative pictures and reduced sustained attention [47]. In our cohort of OSAHS patients, the depressive mood group experienced a reduction in slow-wave sleep and more severe daytime sleepiness, which might be a potential cause of the delayed responses to positive stimuli. Previous research [48, 49] has already demonstrated a higher incidence of depressive mood in patients with OSAHS when combined with daytime Sleepiness. The potential mechanism by which daytime sleepiness in OSAHS patients leads to depressive mood involves long-term sleep fragmentation and intermittent hypoxia. This may lead to the degeneration of the locus coeruleus and arousal-promoting neurons in the mouse brain cortex, resulting in a decrease in the activity of dopamine and norepinephrine neurons, and a reduction in cell count [50, 51]. In summary, our study suggests that the prolonged deprivation of slow-wave sleep in OSAHS patients may be a potential mechanism underlying the development of daytime sleepiness symptoms, which, in turn, could contribute to depressive mood and prolonged N170 latency. Due to the limited sample size, our results could not further assess on the role of daytime sleepiness in the neurophysiological abnormalities in OSAHS patients with coexisting depressive mood. However, we are continuously expanding the sample size, and future research is expected to further analyze the complex relationship between them.

Moreover, a minor finding of our study is that the N170 amplitude in the left occipital lobe was negatively correlated with the arousal index and positively with the MOCA score. This is similar to the findings by Lv et al. in an ERP study using images of facial expressions, which showed an abnormal mismatch negativity (MMN) amplitude associated with MOCA score in OSAHS patients [52]. This could represent a neural compensatory mechanism whereby OSAHS patients counter the disruptive effects of sleep fragmentation on emotional processing. However, a large meta-analysis showed that there is consistent evidence for reduced N170 amplitude only in schizophrenia patients, findings in other diseases have been inconclusive [40].

## Conclusion

Our study showed that OSAHS patients with depressive mood have increased N170 latency and impaired facial emotion recognition ability. These findings highlight the clinical utility of ERP component N170 as a potential biomarker for early identifying depressive mood in OSAHS patients.

### Supplementary Information

**Supplementary Material 1.**

## Data Availability

Access to the dataset utilized in the present study is obtainable upon a reasonable request from the corresponding author.
