# Supplementary material for: Implications of depressive mood in OSAHS patients: insights from event-related potential
Source: BMC Psychiatry. 2024 Apr 23;24:307. doi: 10.1186/s12888-024-05772-6 (PMC11040885; doi:10.1186/s12888-024-05772-6)
Supplement: Supplementary file 1 — Supplementary Material 1. [file 12888_2024_5772_MOESM1_ESM.docx]

Table S1：The Correlations Between N170 Parameters (Amplitude and Latency) and PSG Parameters, as well as Questionnaire Scores

|  | PO7-N170L | PO8-N170L | PO7-N170A | PO8-N170A |
| --- | --- | --- | --- | --- |
| ESS | 0.15961 | 0.17806 | 0.01229 | 0.06361 |
| MMSE | 0.11199 | 0.05259 | 0.08675 | 0.02596 |
| MOCA | -0.06461 | -0.06563 | 0.30376** | 0.08751 |
| BMI | 0.07701 | 0.16773 | 0.15977 | 0.15070 |
| TST | 0.12119 | 0.15088 | 0.01359 | -0.00240 |
| SLEEPEFFI | 0.10607 | 0.15977 | 0.15797 | 0.21882 |
| SLEEPLATENCY | -0.07791 | -0.15445 | 0.03633 | 0.09771 |
| AROUSE | -0.07658 | -0.01897 | -0.47859*** | 0.20830 |
| REM | -0.12744 | -0.19421 | 0.01248 | 0.11726 |
| SWS | -0.17086 | -0.25377 | 0.15644 | 0.06305 |
| ODI | -0.01175 | 0.10258 | 0.03302 | 0.13886 |
| T90 | 0.06102 | 0.13411 | -0.10219 | -0.01118 |
| LSaO2 | -0.01848 | -0.08684 | 0.01878 | -0.03356 |
| AHI | 0.01230 | 0.11395 | -0.05006 | 0.04644 |

Notes: Test level two-sided α=0.05; * *p*<0.05, ** *p*<0.01, ***p*<0.001
